# Transvaginal Expulsion of a Fibroid After Transvaginal Radiofrequency Ablation: A Complication or a Benefit?

**DOI:** 10.3390/reports9020145

**Published:** 2026-05-06

**Authors:** Francesco Cannone, Gianfranco Morreale, Martina Billeci, Ferdinando Antonio Gulino

**Affiliations:** 1 Department of Gynaecology and Obstetrics, Lentini Hospital, Contrada Colle Roggio, SS194, 96016 Lentini, Italy; gianfrancomorreale13@gmail.com (G.M.); martinabilleci31@gmail.com (M.B.); 2 Unit of Gynecology and Obstetrics, Department of Human Pathology of Adults and Developmental Age, University Hospital “G. Martino”, 98100 Messina, Italy; ferdinandogulino@gmail.com

**Keywords:** uterine fibroids, radiofrequency ablation, transcervical treatment, fibroid expulsion, minimally invasive gynecology

## Abstract

Uterine fibroids are among the most common benign tumors affecting women, with a prevalence reaching up to 50–60% in those over 40 years of age, although often underestimated due to asymptomatic cases. Radiofrequency ablation (RFA) represents a minimally invasive alternative to surgery for selected patients. We report the case of a 41-year-old woman with symptomatic uterine fibroids (FIGO type 4, size of 5 cm) treated with transvaginal RFA. One month post- treatment, the fibroid showed partial volume reduction. Two months after the procedure, the patient presented with foul-smelling discharge and heavy bleeding. Ultrasound confirmed complete fibroid migration into the cervical canal. Vaginal removal was performed without complications. Fibroid expulsion after RFA is a rare event that may represent either a complication or a therapeutic outcome. A balanced interpretation and appropriate clinical management are required. Further studies are needed to clarify its clinical significance.

Uterine fibroids, also known as leiomyomas or myomas, are common benign gynecological tumors that affect 50–60% of women aged 40 and over, although the true incidence is likely underestimated due to asymptomatic cases [1,2,3,4,5,6,7,8,9,10,11,12]. In about one-third of cases, they cause symptoms such as heavy menstrual bleeding (hypermenorrhea), dysmenorrhea, dyspareunia, pelvic pain and anemia [2]. The choice of treatment depends on the number, size, and location of the fibroids, as well as the patient’s symptoms and reproductive goals.

Options include expectant management, medical and hormonal therapies, hysteroscopic resection (TCR), laparoscopic or open myomectomy, uterine artery embolization (UFE), and minimally invasive techniques such as radiofrequency ablation (RFA) [3,12,13]. The choice of radiofrequency ablation over uterine artery embolization was based on its minimally invasive nature and its ability to provide targeted treatment while preserving uterine integrity. In this case, the patient preferred a uterus-sparing approach. Moreover, studies suggest that RFA may achieve greater fibroid volume reduction compared to uterine artery embolization, with reported reductions of approximately 60–70% at 6 months versus approximately 50–55% for embolization. RFA is generally indicated for symptomatic fibroids up to 2–11 cm in diameter [4,14]. Transvaginal radiofrequency ablation (RFA) is a technique that allows fibroid treatment without surgical incision. The method uses an ultrasound-guided electrode inserted via the vaginal route into the fibroid tissue. The high-frequency alternating current produces localized heating (60–80 °C), leading to coagulative necrosis, cell death, ischemia, and loss of hormone receptor activity in the treated tissue. And progressive volume reduction [12,14]. Reported volume reduction rates reach approximately 60–70% at 6 months [4,11].

The patient, C.E., is a 41-year-old woman with a history of one spontaneous delivery and prior laparoscopic surgery for endometriosis. She presented to our gynecology unit complaining of hypermenorrhea, pelvic pain, and fatigue due to anemia. Transvaginal ultrasound identified a 5 cm uterine fibroid (FIGO type 4) (Figure 1A). Fibroid volume was assessed using three orthogonal diameters during 2D US. In June 2024, she underwent transvaginal RFA. The procedure was performed via vaginal fornix under ultrasound guidance using (Myoblate). Multiple ablation cycles were applied until adequate coverage of the fibroid was achieved. Intraoperative Doppler assessment demonstrated a reduction in vascularization after treatment.

The procedure was completed without intraoperative or postoperative complications. One month follow-up, the patient reported improvement in pelvic pain but continued to experience heavy menstrual bleeding. Ultrasound showed the fibroid had decreased by approximately 1 cm (Figure 1B). Two months after the procedure, she returned with complaints of excessive hypermenorrhea, spotting, smelly vaginal discharge, and pelvic pain. Transvaginal ultrasound revealed the fibroid had migrated into the cervix with a vascular pedicle (Figure 1C). On gynecological examination, the fibroid was visible at the cervical level. A colposcopic view was used to better document the lesion and obtain a clear image for illustrative purposes (Figure 1D).

Given the risk of infection associated with necrotic tissue, antibiotic therapy was administered with 2 g of ceftriaxone. No signs of systemic infection were observed. vaginal removal of the fibroid was performed under general anesthesia. The fibroid was grasped with forceps, and gentle torsion was applied to the pedicle until complete detachment was achieved. No additional cutting instruments were required. The procedure was completed without significant bleeding, and no uterine curettage was necessary, as no residual tissue was identified. The procedure lasted approximately 10 min and was completed without complications. (Figure 1E). The patient was discharged in good health. Histopathological analysis confirmed a benign leiomyoma.

The mechanism of fibroid expulsion after RFA is not fully understood. It is likely related to thermal-induced coagulative necrosis, leading to ischemia, inflammatory response, and uterine contractions that promote detachment of the fibroid. In cases with submucosal components, the necrotic tissue may migrate into the uterine cavity and be expelled through the cervix.

Fibroid expulsion is a rare event, with an estimated incidence of approximately 1–1.5%, and has also been described after uterine artery embolization [10].

Fibroid expulsion is a rare post-RFA event. To date, only one similar case has been reported, from a hospital in Germany in 2023. Common symptoms of fibroid expulsion include vaginal bleeding, smelly discharge, and the sensation of a mass passing through the vagina. Fibroid expulsion following RFA may represent a double-edged outcome. On one hand, it may allow complete removal of the fibroid through a minimally invasive approach. On the other hand, it may be associated with complications such as infection, bleeding, or the need for intervention. Therefore, it should be considered a post-procedural event requiring careful monitoring rather than strictly a complication or a benefit.

In this case, the outcome was favorable, with complete fibroid removal and symptom resolution.

Further studies are needed to better define the mechanisms, incidence, and clinical implications of fibroids.

## Figures and Tables

**Figure 1:**
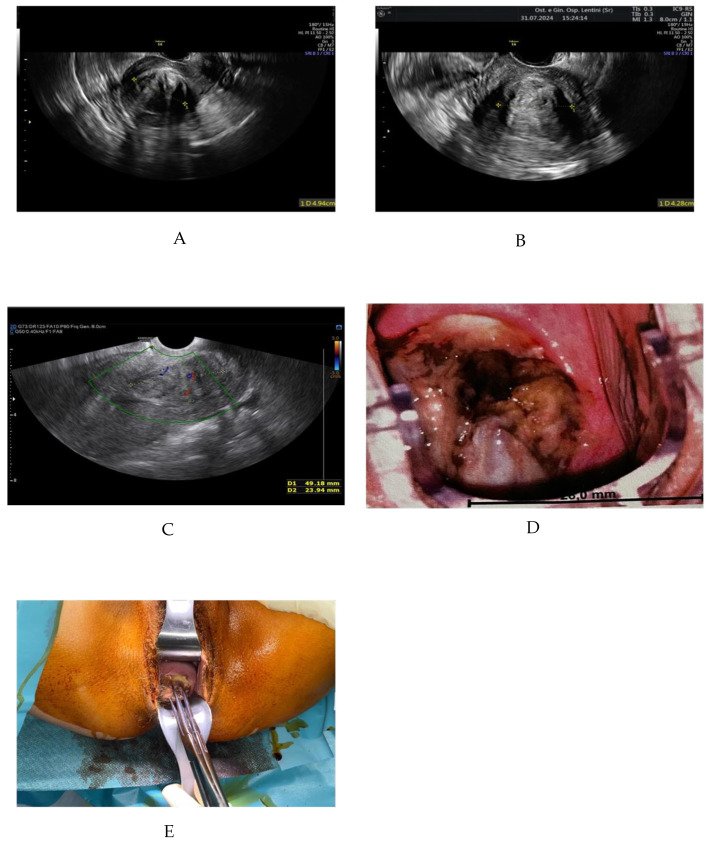
(**A**) Transvaginal ultrasound showing a 5 cm FIGO type 2–5 fibroid before RFA; (**B**) One month post-RFA, showing a reduction in fibroid size; (**C**) Two months post-RFA, Doppler ultra- sound revealing fibroid descent into the cervical canal; (**D**) Cervical fibroid visualized during colposcopic examination (28.0 mm); (**E**) Intraoperative image showing the removal of the expelled fibroid.

## Data Availability

The original contributions presented in this work are included in the article. Further inquiries can be directed to the corresponding author.
